# Measurement of pressure dependent variations in local pulse wave velocity within a cardiac cycle from forward travelling pulse waves

**DOI:** 10.1038/s41598-025-87143-z

**Published:** 2025-01-24

**Authors:** Rahul Manoj, Kiran V. Raj, P. M. Nabeel, Mohanasankar Sivaprakasam, Jayaraj Joseph

**Affiliations:** 1 Department of Electrical Engineering, Indian Institute of Technology Madras, Chennai, India; 2 Healthcare Technology Innovation Centre, Indian Institute of Technology Madras, Chennai, India

**Keywords:** Pulse wave velocity, Pressure-dependent pulse wave velocity, Wave separation analysis, Pulse transit time, Wave reflection, Carotid artery, Cardiovascular biology, Biomedical engineering, Predictive markers

## Abstract

The local pulse wave velocity (PWV) from large elastic arteries and its pressure-dependent changes within a cardiac cycle are potential biomarkers for cardiovascular risk stratification. However, pulse wave reflections can impair the accuracy of local PWV measurements. We propose a method to measure pressure-dependent variations in local PWV while minimizing the influence of pulse wave reflections. The PWV is computed from the pulse transit time between two forward-traveling pulse waveforms obtained across known path length, after measured/modelled flow-based wave separation analysis (WSA). An in-vivo study of 60 participants (24 female), was conducted to compare inter- and intra-cycle variations in PWV obtained from measured and forward pulse waves. For this, proximal and distal diameter waveforms from the carotid artery, along with carotid tonometry, were recorded using a custom bi-modal arterial probe. The carotid blood flow for WSA was captured with an ultrasound imaging system. The reference PWV was derived from the Bramwell-Hill equation. After WSA, the reliability of PWV measurement improved with coefficient of variation reducing from 25% to 10% near the peak of the pulse waves and matched the reference PWV with no statistically significant difference. The average PWV at foot of the pulse wave before and after WSA were comparable to the reference PWV with no statistically significant difference. The coherence of carotid pulse pressure obtained from the mean values of PWV within a cardiac cycle after WSA with that of the carotid pulse pressure from tonometry, substantiates the results obtained for reflection-free PWV. The reliability of measuring local PWV and its pressure dependent variations within a cardiac cycle is improved by combining transit-time approach with WSA.

## Introduction

**Fig. 1:**
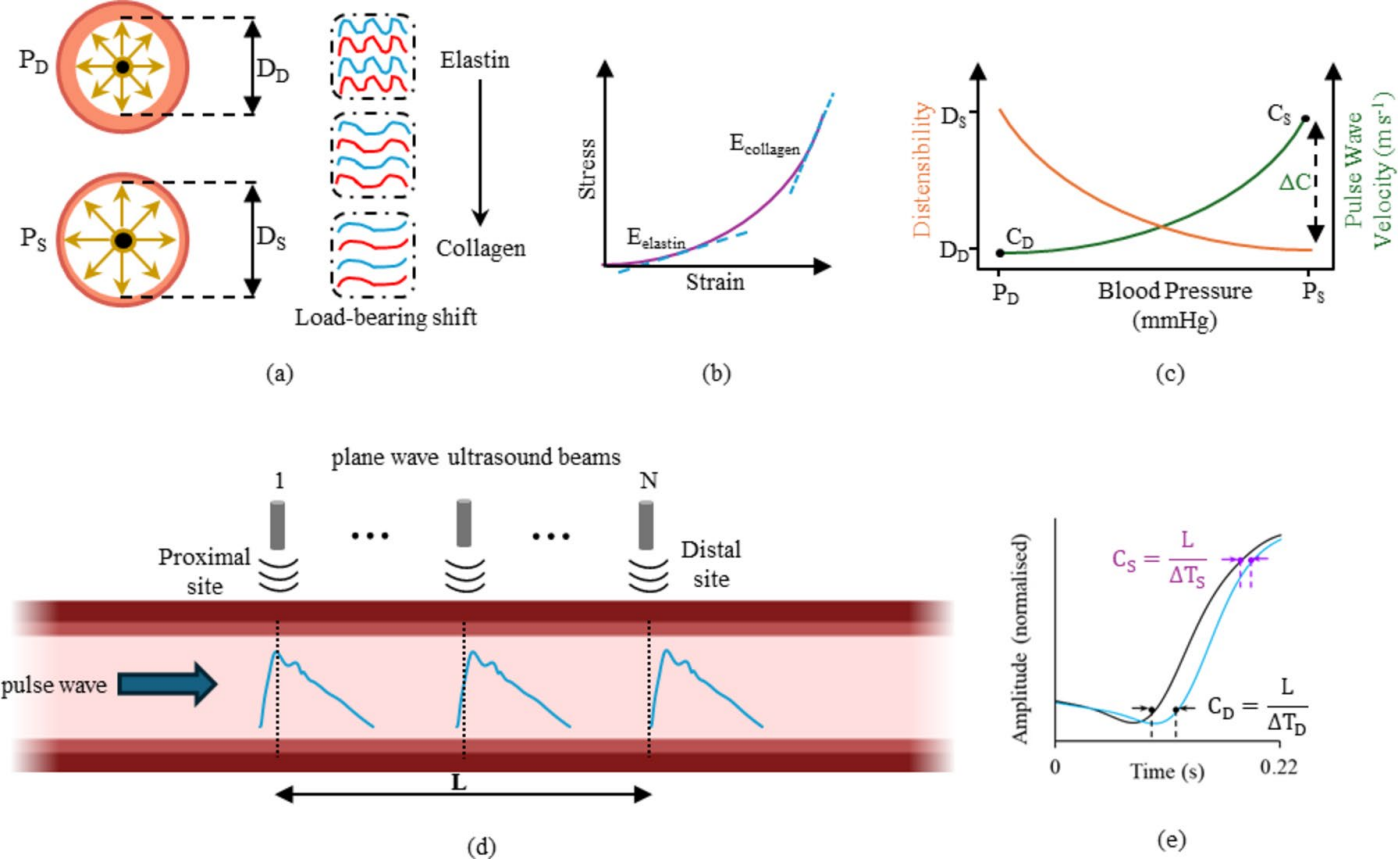
(a) Load-bearing shift within a cardiac cycle from elastin to collagen as the artery distends under pulsatile pressure conditions (P_D_ – diastolic blood pressure, P_S_ – systolic blood pressure) and distension (D_D_ – diastolic diameter, D_S_ – systolic diameter), (b) Heterogenic nature of the artery vessel wall produces an incremental elastic modulus, primarily due to the load bearing shift from elastin (E_elastin_ – modulus of elasticity of elastin) to collagen (E_collagen_ – modulus of elasticity of collagen) within a cardiac cycle, (c) the pressure-dependent variations in pulse wave velocity (ΔC) from diastole (C_D_) to systolic (C_S_) during the anacrotic phase of cardiac cycle, (d) plane wave imaging of N ultrasound beams recording the arterial pulse wave simultaneously, (e) spatiotemporal wave propagation and measurement of pulse wave velocity using transit time method, L – length of the artery from proximal measurement site to distal measurement site, ΔT_D_ and ΔT_D_ corresponds to the pulse transit time near diastole and systole respectively.

The heart’s ejection of a blood volume into the aorta, creates a travelling pulse wave through the compliant blood vessel wall. The measure of the velocity of the travelling pulse wave, called the pulse wave velocity (PWV) is a clinically recognized marker of arterial stiffness^1^. The PWV, when measured over large trajectories of anatomical length such as carotid-to-femoral or brachial-to-ankle are referred to as regional PWV, and those measured local to a specific arterial site are referred to as local PWV. The regional PWV provides an average estimate of the whole-body vascular stiffness, whereas the local PWV measures the local stiffness of specific arteries. Conventionally, the local PWV is measured from the foot of the pulse cycle, yielding a value at the diastolic blood pressure (DBP). However, with measurements of higher spatiotemporal resolutions, local PWV at various fiducial points within the systolic phase at various BP is now measurable^2^. This change in PWV from diastolic BP to systolic BP within a cardiac cycle is called incremental PWV (ΔC)^2^. The vessel wall with its heterogenic and layered material composition by endothelial cells, elastin, smooth muscle cells and collagen fibers produces a non-linear incremental stress-strain behavior^3^. The PWV is closely associated with the material properties of the arterial wall and wall tension^4^, which change dynamically throughout the cardiac cycle. This behavior results in pressure-dependent variations in PWV within a cardiac cycle^5–7^ as illustrated in Fig. 1(a)-(c). The changes in local PWV and ΔC are markers of early manifestation of cardiovascular diseases and therefore are essential tools for assessing early vascular ageing (EVA). The assessment of EVA non-invasively from the arterial pulse waveform marks the next generation of preventive care tools for cardiovascular risk stratification and mitigation of the disease burden^8^.

### Clinical significance

The central blood pressure (cBP) being the blood pressure (BP) directly applied on vital organs by aorta and its direct branches, has superior diagnosis for hypertension and EVA over brachial blood pressure (bBP)^9–11^. The limited usage of cBP in routine clinical examination is often due to lack of non-invasive technologies for measuring cBP, in comparison to auscultation or oscillometery BP devices^12^. To circumvent this situation, left common carotid arty (CCA) is a preferred alternate arterial site often used as a surrogate for central hemodynamics, due to the direct branching to the aorta and anatomical advantage around the neck region to accommodate non-invasive sensing modalities^12^. Measurement of arterial stiffness and cBP at CCA using local PWV and ΔC offer means to develop non-invasive instruments for measuring central hemodynamics and are vital in understanding the pathophysiologic mechanisms of EVA. Recent studies have demonstrated that measuring the local PWV at the CCA serves as a prognostic indicator for atherosclerosis^13^, stroke^14^and coronary heart disease^15^. Furthermore, independent studies have notably shown that ΔC is effective in identifying various cardiovascular changes, including damage to blood vessels caused by hypertension^16^, myocardial hypertrophy due to increased load^6^, and Vascular Ehlers-Danlos syndrome^17^, beyond traditional risk markers. Recent population-level studies^7^have emphasized the prognostic ability of ΔC in predicting cardiometabolic risks. The measurement of ΔC also serves as the basis for computing cBP in a calibration-free approach^18,19^, using non-invasive technologies that can be readily translated to clinical practice.

### Methodological challenges

Measurement of local PWV is categorized as single-point measurements, dual-site measurement and multi-site measurement^2^. Theoretically, PWV is derived from solutions to 1-D Navier-Stokes equations for pulsatile blood flow in an elastic vessel, and expressed as Moens-Korteweg (MK) and Bramwell-Hill (BH) Eq^4^. Recent numerical studies modify the MK and BH equations to accommodate additional non-linearity^20,21^as well. Single-point measurements requires a linear region between measured hemodynamic variables – pressure (P), blood flow rate (Q), blood flow velocity (U), vessel diameter (D) and cross-sectional area (A) of vessel to compute the PWV. The linear region is the slope of the constructed hemodynamic-loops – PU loop, QA loop, ln(D)U and DP loop in the anacrotic phase of a cardiac cycle which is assumed to be devoid of any effects of pulse wave reflections, to estimate PWV^2^. To construct a hemodynamic loop, there is a requirement of a pair of pulse waveforms – P-U, Q-A or D-U, D-P which needs to be obtained from the same arterial site simultaneously. Single-point measurements fundamentally limits the measurement of PWV to a single-value or an average-value estimate impeding the measurement of ΔC.

Both single-point, dual site or multi-site measurements for PWV may potentially be subjected to time-lags between the hemodynamic signal pairs while recording or processing. Among the single-point loop-based approaches, this time-lag will affect the linear relationship between hemodynamic signal pairs which is the basic underlying assumption and corrupt the estimate of local PWV^2^. For instance, the PU loop is constructed from carotid tonometry and doppler ultrasound, QA and ln(D)U from the duplex mode of ultrasonography. The use of different modalities to record a pair of pulse waveforms could introduce time-lags due to their mismatches in frequency response^22,23^. In all the above cases, an additional time-axis alignment either with a reference ECG or with respect to the foot of the pulse wave by visual inspection becomes essential. The accuracy of post processing of video files for measuring the D, A, Q and U from ultrasonography is limited by the low frame rates of ultrasonography machines (~20 to 40 Hz) and from the pixel-scaling calibration when tracing out the pulse waveform. The simultaneous measurement of pulse waveform pairs is another challenge due to the form-factor of commercially available transducers, leading to time-lag due to positional offsets. This limitation arises due to the current commercial ultrasound system being made for generic imaging purposes only. With recent advancements in ultrasound and other sensing modalities^23,24^, this is not a fundamental limitation in the future. Lastly the signal processing algorithms used for filtering and fiducial point identification may potentially shift the waveforms, introducing additional time-lags^25^. Therefore, both single-point, dual site or multi-site measurements for PWV requires incorporation of effective time synchronization algorithms such as zero-phase digital filters to hardware-synchronized simultaneous acquisition of pulse signals to compensate for the additional time-lags that are potentially introduced^23^.

It has been experimentally shown that wave reflections are present even as close as the systolic foot of the pulse waves^26^. Therefore, the assumption of a reflection-free linear region in the early upstroke of the pulse waves can be misleading^1,2^. The inconsistencies in the values of PWV observed among the various single-site methodologies is also attributed to the influence of pulse wave reflections^27,28^. The most direct measurement of PWV is computed from the spatiotemporal wave propagation using the dual or multi-site techniques^2^. Figure 1(d), (e) provides an illustration on spatiotemporal wave propagation and measurement of PWV. Essentially, the method involves computing the pulse transit time (PTT) as the time delay between simultaneously recorded pulse waves from two or more sites, with known path length between each measurement site. The measurement of PWV using the dual or multi-site techniques largely depends on the accurate description of the waveform morphology and the waveform features derived from it. The waveform features from both systolic peak^29,30^and foot^26^ are influenced by pulse wave reflections, modifying the pulse morphology, hindering the reliability of measuring local PWV and its pressure dependent variations within a cardiac cycle. Subsequent section elaborates more on the methodology we propose to improve the reliability of measuring local PWV.

**Fig. 2:**
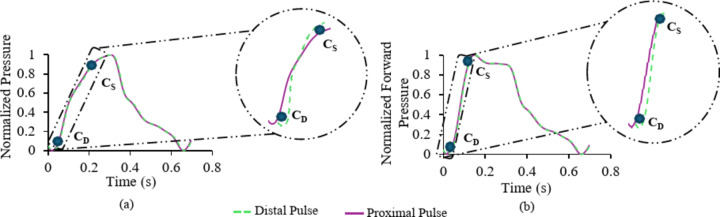
(a) Methodological constraints on the assessment of pulse transit time from two simultaneously recorded and normalized pulse waveform obtained from common carotid artery illustrating the effect of pulse wave reflections, hindering the measurement of pulse wave velocity (b) pulse transit time calculated from normalized forward-travelling pulse waves after minimizing the effects of pulse wave reflections using wave separation analysis. C_S_ denotes the pulse wave velocity near systole and C_D_ denotes the pulse wave velocity near diastole.

### Proposed solution and validation strategies

In this work, we tested the hypothesis that quantification of arterial wave reflection and separation of a pulse wave into forward and backward waves is essential to improve the measurement accuracy and precision of pressure dependent dynamic changes in local PWV. The PWV calculated from the PTT of forward-travelling waves will potentially improve the reliability of the PWV measurement. A similar approach for improved estimation of carotid-to femoral PWV was demonstrated using arterial tube-load model-based wave separation algorithms^31^, however, the applicability to local PWV or its validation with theoretical PWV was not shown. Conventional wave separation analysis (WSA) using measured or modelled flow has been widely applied to aortic site^32^, however, in this work, we apply WSA to signals recorded from CCA, and further propose the application of a pressure-only WSA tailored for CCA using a multi-Rayleigh flow model which is previously validated on in-silico virtual subjects^33^and in-vivo studies^34^. Figure 2 illustrates the computation of PWV using transit-time methods before WSA and after WSA. The primary objective of this study is to validate methods that can improve the reliability of measuring local PWV and ΔC at the CCA, by minimizing the effect of pulse wave reflections that are hindering the accuracy and precision of measurement. Specific objectives and validation strategies include:

1. To perform WSA using both measured flow (WSA_REF_) and modelled flow (WSA_m-RAY_)^33,34^ at CCA to separate the forward and backward travelling waves.
2. To compute PWV using BH Eq. 4 as a reference measurement of theoretical estimate, using carotid tonometry calibrated pressure waveform and diameter waveform.
3. To compute and compare the PWV near the foot and peak of the pulse wave using PTT-based method on two simultaneously recorded pulse waves before WSA and from forward-travelling pulse waves after WSA.
4. To evaluate the coherence in the estimated carotid pulse pressure (ΔP) obtained from mean values of PWV obtained before WSA and after WSA, using the theoretical expression, with that of ΔP obtained from tonometry-calibrated pressure waveform. The obtained carotid ΔP is also compared with aortic ΔP from SphygmoCor^®^ XCEL and brachial ΔP from oscillometery devices.

## Methods

### Principle of measurement of reflection-free PWV

The PWV estimation based on temporal and spatial gradients is a direct way to assess how fast the pulse wave propagates along the artery by tracking changes in the pulse waveform^2^. Mathematically, time-dependent variation of PWV within a cardiac cycle (C(t)) is expressed as the ratio of temporal gradient to spatial gradient^35–37^ of the generic pulse waveform f(t) as in Eq. (1)

1 $$\:\begin{array}{c}C\left(\text{t}\right)=\frac{\dot{{\text{f}}_{\text{t}}}(\text{t},\text{x})}{\dot{{\text{f}}_{\text{x}}}(\text{t},\text{x})}\end{array}$$

Where, $\:\dot{{\text{f}}_{\text{t}}}(\text{t},\text{x})$ is the temporal gradient of the travelling pulse waveform, and $\:\dot{{\text{f}}_{\text{x}}}(\text{t},\text{x})$is the spatial gradient of the pulse waveform, along the axis of the artery. In most literature, the measurement of PWV is limited to a definite fiducial point (τ, L). The two-point direct measurement of PWV (C) is measured as the ratio of known anatomical length (L) between proximal and distal measurement sites to the pulse transit time (PTT) between two simultaneously recorded pulse waves^2^ as in Eq. (2).

2 $$\:\begin{array}{c}C=\:\frac{\text{L}}{\text{P}\text{T}\text{T}}\end{array}$$

Equation (2) is a special case of (1), where (t, x) are (τ = PTT at a specific fiducial point, L). Accounting for the non-linear material property and wall tension that changes dynamically within the cardiac cycle, C(t) takes the form for incremental PWV (ΔC) – pressure dependent intra-cycle variations in PWV within a cardiac cycle. Therefore, Eq. (1) is re-written as,

**Fig. 3:**
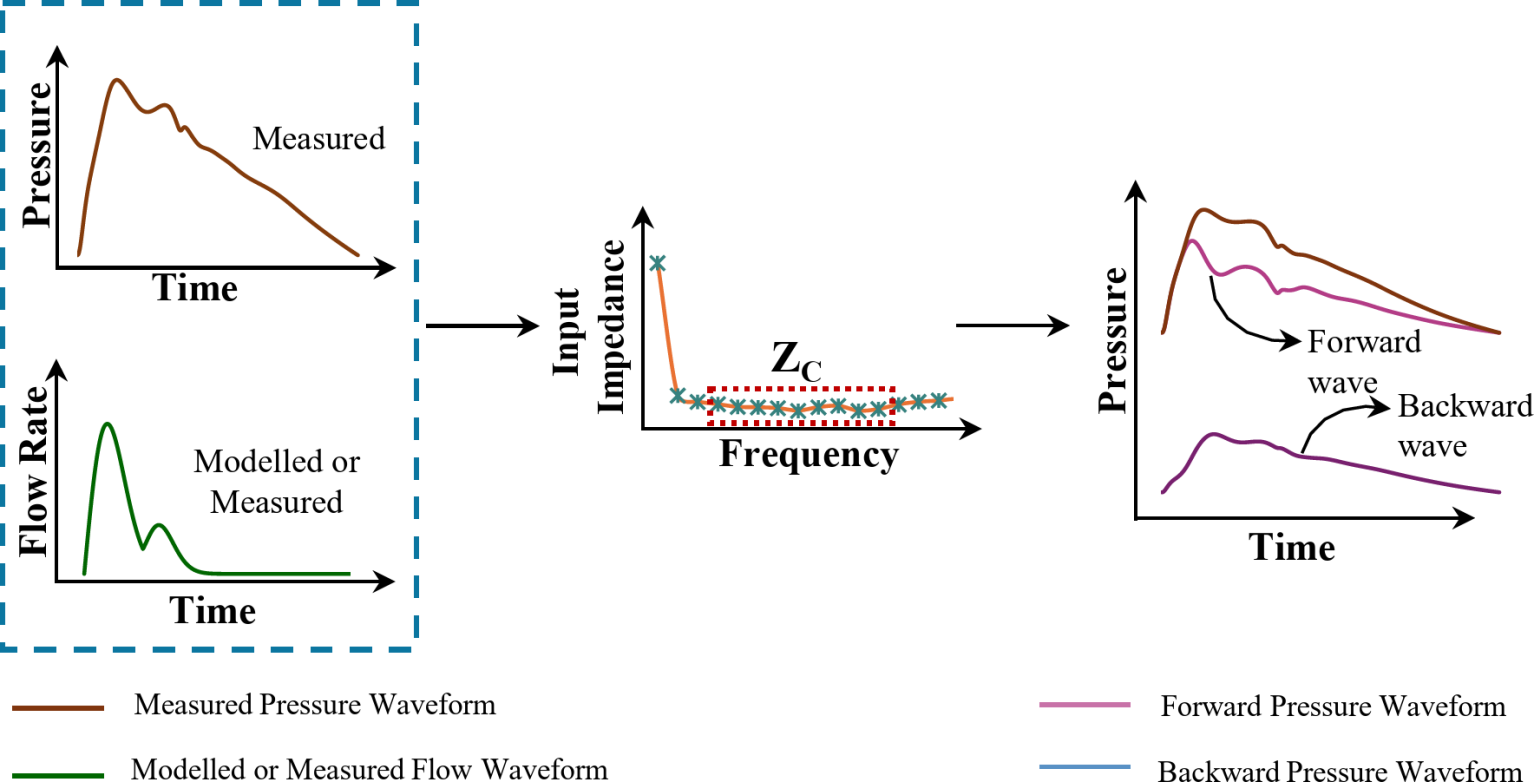
Overview of wave separation analysis using measured pressure waveform and measured or modelled flow waveform to decompose a pulse waveform into its forward and backward components.

3 $$\:\begin{array}{c}\begin{array}{c}{\text{C}}_{k}=\frac{\dot{{\text{f}}_{\text{t}}}({\text{t}}_{\text{k}},\text{x})}{\dot{{\text{f}}_{\text{x}}}({\text{t}}_{\text{k}},\text{x})}\end{array}\end{array}$$

Where C_k_ is C_D_ – PWV closer to foot of the pulse wave, C_M_ – PWV at the mean level of the pulse wave and C_S_ – PWV closer to the peak of the pulse wave, corresponding to PTT measured at each of the levels and $\:{\text{t}}_{\text{k}}$ varies from the foot of the pulse wave to the peak of the pulse wave. Equation (3) takes a simplified form for two pulse waves, recorded simultaneously with a known distance L and with PTT of ΔT_D_ and ΔT_S_ at points closer to diastolic and systolic as

4 $$\:\begin{array}{c}\begin{array}{c}{\text{C}}_{\text{D}}=\frac{\text{L}}{\varDelta\:{\text{T}}_{\text{D}}}\end{array}\end{array}$$

5 $$\:\begin{array}{c}\begin{array}{c}{\text{C}}_{\text{S}}=\frac{\text{L}}{\varDelta\:{\text{T}}_{\text{S}}}\end{array}\end{array}$$

and ΔC = C_S_ – C_D_^7,18^. The reflection-free PWV is computed from the forward-travelling components of the simultaneously recorded pulse waves from both the measurement sites. The WSA decomposes the recorded proximal and distal pulse waves into its forward and backward components. The wave separation theory is derived by combining the water hammer equations with transmission line circuit analogy^38^, as

6 $$\:\begin{array}{c}{\text{P}}_{\text{F},\text{p}\text{r}\text{o}\text{x}}\left(\text{t}\right)={\text{P}}_{\text{p}\text{r}\text{o}\text{x}}\left(\text{t}\right)+{\text{Z}}_{\text{C}}Q\left(\text{t}\right)\end{array}$$

7 $$\:\begin{array}{c}{\text{P}}_{\text{F},\text{d}\text{i}\text{s}\text{t}}\left(\text{t}\right)={\text{P}}_{\text{d}\text{i}\text{s}\text{t}}\left(\text{t}\right)+{\text{Z}}_{\text{C}}Q\left(\text{t}\right)\end{array}$$

where, $\:{\text{P}}_{\text{p}\text{r}\text{o}\text{x}}\left(\text{t}\right)$and $\:{\text{P}}_{\text{d}\text{i}\text{s}\text{t}}\left(\text{t}\right)$ are the simultaneously recorded pulse waveforms. Q(t) is the flow waveform measured or modelled from the same arterial site. $\:{\text{P}}_{\text{F},\text{p}\text{r}\text{o}\text{x}}\left(\text{t}\right)$ and $\:{\text{P}}_{\text{F},\text{d}\text{i}\text{s}\text{t}}\left(\text{t}\right)\:$are the derived forward components of respective pulse waveforms. $\:{\text{Z}}_{\text{C}}$ is the estimated characteristic impedance of the arterial vessel. Figure 3 illustrates an overview of WSA using measured pressure and modelled or measured flow waveform to separate into forward and backward wave components.

### Multi-rayleigh flow model for wave separation analysis

**Fig. 4:**
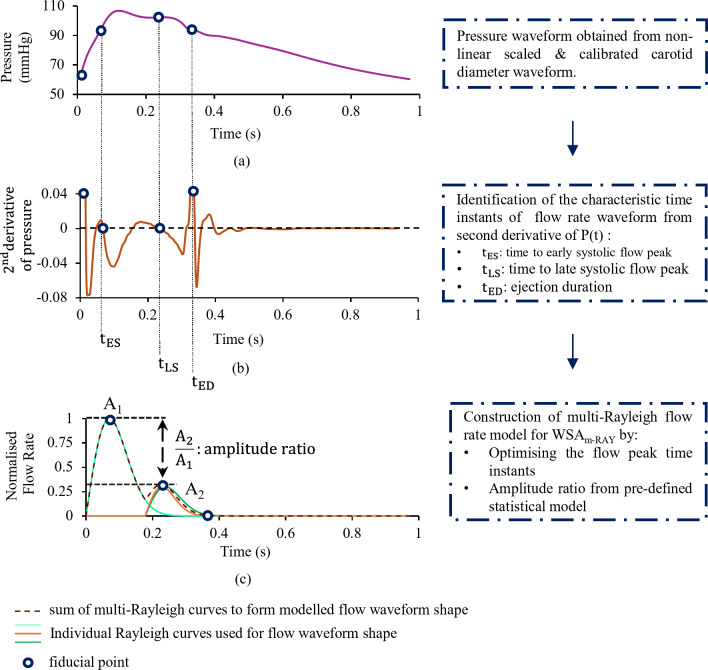
Construction of the multi-Rayleigh flow shape waveform for wave separation analysis (WSA_m−RAY_) based on the optimization of time instants of flow peaks – obtained from the second derivative waveform and amplitude ratio of flow peaks using a pre-defined statistical model that correlates flow augmentation with pressure augmentation.

The WSA_m−RAY_is a validated technique to perform forward-backward wave separation at CCA^33,34^. A flow morphology approximation is constructed using weighted and shifted Rayleigh functions that reliably models the time instants of early systolic flow peak and late systolic flow peak. A loss function is defined from the time instants of the flow peaks derived from fiducial points obtained from higher derivative waveform of the recorded pressure or diameter cycles at CCA and with that of the time instants of modelled flow peaks. An iterative algorithm optimizes the number of multi-Rayleigh curves that are needed to minimize the loss function. The amplitude ratio of early systolic flow peak to late systolic flow peak is derived from an exponential regression model, between the reference flow peak amplitude ratio (A_2_/A_1_) and Augmentation Index (AIx). Figure 4 depicts major steps involved in the construction of the modelled flow waveform for WSA_m−RAY_. A detailed description of the model, its verification and validation in both in-silico and in-vivo studies may be referred to elsewhere^33,34^. For WSA, the magnitude of the flow waveform is irrelevant, as the product of Z_C_ and Q(t) is ratio metric, evident from Eqs. (6) and (7). However, in this study the peak of the modeled flow is scaled to its known magnitude from the measured flow.

### Instruments for data collection

The BP from the brachial artery (bBP) is recorded using a bladder-cuff type oscillometery device (SunTech^®^ 247, USA). The aortic BP is estimated using SphygmoCor^®^XCEL based on the generalized transfer function-based approaches on peripheral pulse waveforms recorded from a bladder-type cuff wound on upper left arm. The CCA blood flow is recorded as Doppler flow velocity obtained using clinical grade ultrasound imaging system (Sonix Touch+, BK Medicals, USA). The imaging system consists of a linear array probe (center frequency: 10 MHz, pitch: 0.3 mm, depth: 4 cm). The Duplex-Mode with pulsed wave Doppler was used to record continuous flow velocity waveforms as a video file in .avi format at a frame rate of 40 Hz. The recorded video files were converted into time series flow velocity waveforms using an edge-detection based open-sourced software^39^ in MATLAB^®^ and saved as .csv files for further analysis.

**Fig. 5:**
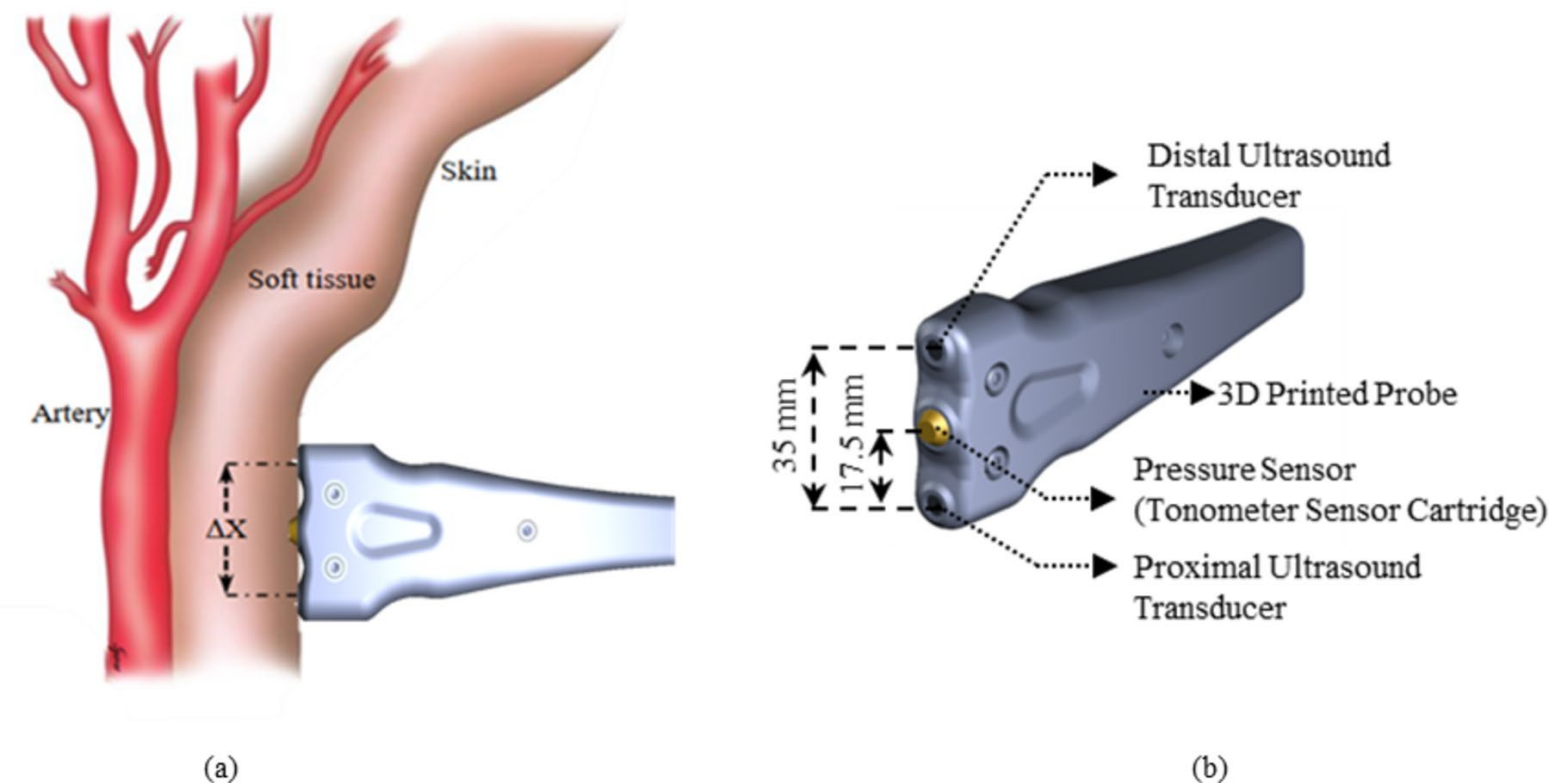
(a) Positioning of the in-house developed bi-modal arterial probe with respect to common carotid artery, (b) parts of the bi-modal arterial probe.

The dual diameter waveforms from two locations on the CCA – proximal and distal, along with tonometry pulse cycles are recorded using an improved version of the in-house developed bi-modal arterial probe^23^. The bi-modal arterial probe consists of two custom-made focused ultrasound transducers (center frequency: 5 MHz, diameter: 5 mm, spatial angle < 1.3^◦^) separated by a center-to-center distance of 35 mm. A custom-made tonometer sensor cartridge (Millar Instruments, USA, identical to SPT-301 Tonometer, Sensitivity: 5µV/V/mmHg, Range: −50 mmHg to 300 mmHg) is placed at the center, equidistant to both ultrasound transducer. Both tonometer and ultrasound transducers are housed in an ergonomically designed 3D-printed probe as depicted in Fig. 5. The radio frequency (RF) echoes from the ultrasound transducers are simultaneously recorded along with the tonometry pulse cycles in a synchronized framework for beat-to-beat evaluation of processed pressure and diameter cycles. The details on the hardware and software architecture of the bi-modal probe are referred to elsewhere^23^.

### Study design and protocol

An observational cross-sectional study was conducted on 60 participants (24 female), aged 20 to 60 years. The study was conducted at Indian Institute of Technology Madras, India. The study design and protocol were approved by Institute Ethics Committee (IEC/2021-01/JJ/07). All the participants are informed about the study, and they have all signed the informed consent form. The study design and protocol for data collection strictly adheres to the principles laid out in.

the latest guidelines of the of the Declaration of Helsinki for medical research involving human subjects. Inclusion criteria include male and female participants above age 18 years and less than 86 years. Participants with documented evidence of carcinoma, human immunodeficiency virus infection, or severe psychiatric illness are excluded. Pregnant women, pediatric or elderly people and patients with arrhythmia, unstable clinical situation, high-grade stenosis of the carotid artery, and carotid sinus syndrome are also excluded from the study.

**Fig. 6:**
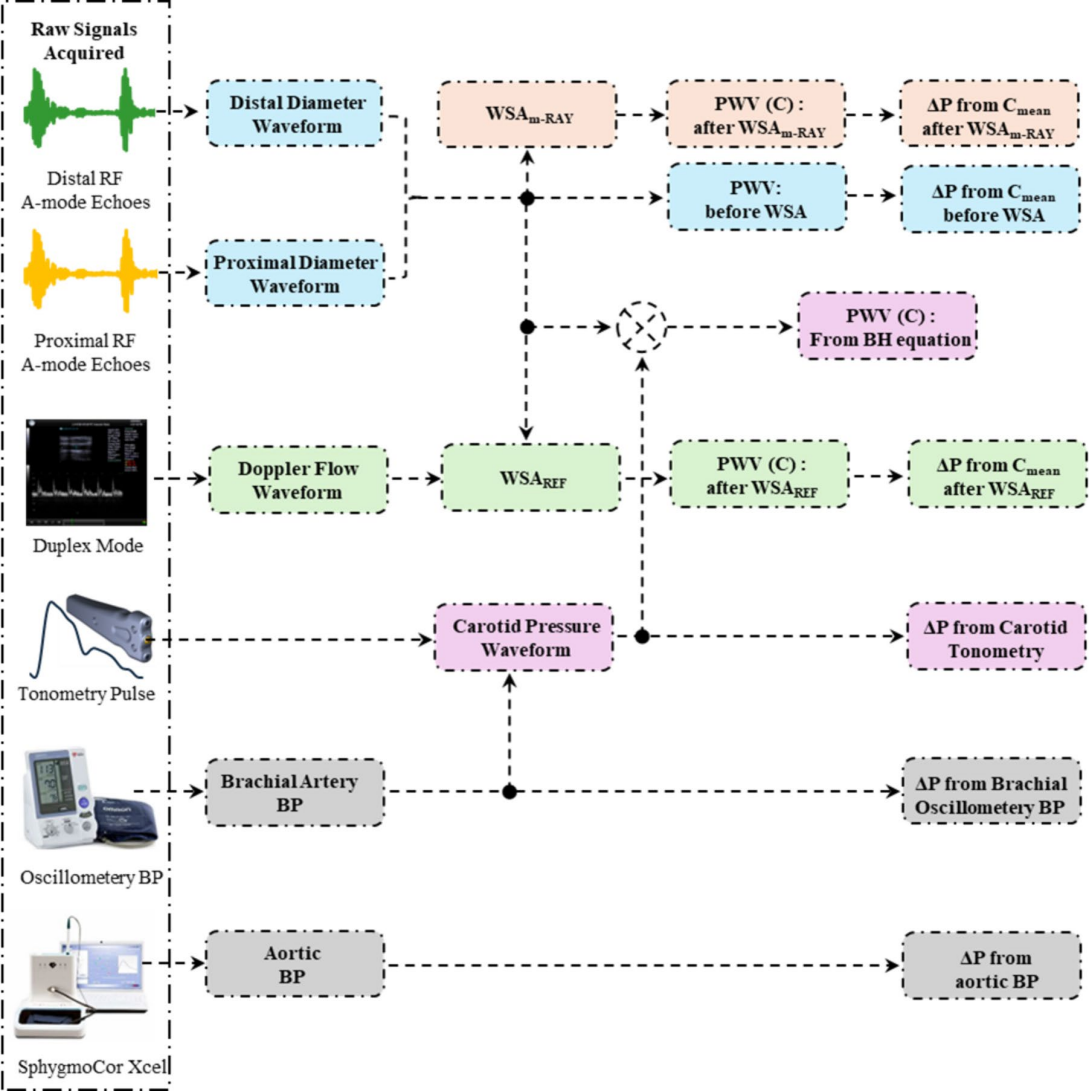
Instrumentation flow chart for raw hemodynamic signals acquisition and data processing involved. WSA denotes wave separation analysis, WSA_m−RAY_ denotes multi-Rayleigh based WSA, ΔP denotes pulse pressure, BP denotes blood pressure.

Participants were required to fill out a detailed questionnaire regarding their medical and genetic backgrounds, any medications presently being taken, dietary habits, consumption frequency of tobacco and alcohol. Anthropometric measurements which include height, weight, and Body Mass Index (BMI) were recorded from each participant prior to the measurements. The study took place in an environment with controlled temperature settings, maintained between approximately 24 °C and 26 °C, where participants were asked to remain in a supine position for the duration of the study. The assessment commenced with the measurement of bBP using a cuff-based oscillometery device (SunTech^®^ 247, USA) on the participant’s upper left arm. The average systolic blood pressure (SBP), diastolic blood pressure (DBP), and mean arterial pressure (MAP) were computed from three repeated BP recordings.

A flowchart illustrating the various measurements performed on the participants is referred to Fig. 6. A linear array probe of a clinical-grade (Sonix Touch+, BK Medicals, USA) ultrasound imaging system was positioned and oriented on the participant’s neck to secure a clear longitudinal cross section of the left common carotid artery (CCA) in the Duplex-Mode. An appropriate gate width (approximately one-third of the lumen diameter) and probe angle were adjusted to accurately measure the Doppler blood flow velocity, reliably capturing the tri-phasic nature of CCA blood flow pattern. The video file in duplex-mode is recorded for a period of thirty seconds and saved. The bi-modal arterial probe was positioned and oriented in the neck region of the participant, until sharp A-mode ultrasound RF echoes were received from both the near and far walls of the CCA from both proximal and distal measurement site. The tonometry pulse cycles were recorded using the same probe, under slight applanation. Continuous A-mode RF signals along with the tonometry pulse waveforms were recorded over a span of thirty seconds. To ensure the selection of the highest quality waveforms for analysis, multiple thirty-second recordings were conducted and the one with the highest signal quality (SQ) index^40^ is used. The SQ index for each ultrasound transducer is calculated in real-time based on the signal echo sharpness and energy, which are higher when the artery is perpendicular to the transducer. The echo signals from the transducers are only recorded when the SQ is beyond a certain threshold, which indirectly confirms that the artery’s orientation is close enough to the assumed perpendicularity with the ultrasound beam for reliable measurements. The aortic BP estimation was recorded using the bladder-type cuff wound on the participant’s upper left arm using the SphygmoCor^®^ XCEL device. All the measurements were collected in a sequential manner. To ensure that the participants’ hemodynamic conditions remained stable throughout the study, oscillometery BP was measured at the end of the study. This final measurement was compared with initial values to confirm that BP remained within the range established at the beginning of the study.

### Data processing

The digitized RF echoes from proximal and distal sites were analyzed to identify the vessel and tracked frame-to-frame to arrive at the respective diameter waveform using validated tracking algorithms^41^. The proximal and distal diameter waveforms were scaled non-linearly based on Hayashi-Kawasaki pressure – diameter exponential relationships and calibrated with brachial MAP and DBP to obtain respective diameter-calibrated pressure waveforms^42^. The tonometry pulse waves were linearly calibrated with brachial MAP and DBP to obtain CCA pressure waveform. The Duplex-Mode video files were processed using open-source software to trace out the flow waveform morphology as a time series signals. The traced signals were calibrated with peak systolic and end-diastolic values of flow velocity obtained from the saved video files. The flow rate waveform was obtained by combining the flow velocity and cross-sectional area of the blood vessel. Diameter, tonometry-pressure, and flow rate waveforms underwent the identical zero-phase low pass Butterworth filtering (cutoff: 15 Hz, order: 3) to preserve the signal integrity across the waveforms and were up sampled to 10 kHz. The sequentially obtained flow waveform is time-aligned with the simultaneously obtained proximal, distal diameter and tonometry-pressure waveforms by adjusting to the end-diastolic foot of all the waveforms. Five continuous cycles were used to compute time-averaged cycles for WSA and PWV calculation.

The study participants were classified into Type-A, Type-B and Type-C waveforms based on the AIx calculated from the pulse wave analysis on diameter-calibrated pressure waveforms^43^. The proximal and distal diameter cycles were scaled from 0 to 1 for measuring the PTT and PWV using Eqs. (4) and (5). The time instant corresponding to 10% and 80% from the end-diastolic foot of both the proximal and distal pulse waveforms were calculated, and the temporal difference between them was measured as the ΔT_D_ and ΔT_S_ respectively within a cardiac cycle, as illustrated in Fig. 1(e). The corresponding C_D_ and C_S_ were calculated using Eqs. (4) and (5), illustrated in Fig. 2(a). However, for reflection-free PWV, the forward-travelling pulse waves were derived from WSA for both proximal and distal sites, followed by the same procedure of PTT measurement as described, illustrated in Fig. 2(b). The carotid ΔP is calculated from the mean values of PWV (C_mean_) obtained before and after WSA, combined with the BH Eq. 4 as in Eqs. (8),

8 $$\:\begin{array}{c}\varDelta\:P=\:2\rho\:\times\:{\text{C}}_{\text{m}\text{e}\text{a}\text{n}}^{2}\times\:\frac{\varDelta\:\text{D}}{{\text{D}}_{\text{D}}}\end{array}$$

Where, ρ is the density of blood, ΔD is the maximum wall distention, and D_D_ is the end-diastolic lumen diameter of the carotid artery.

The algorithm for modelling the flow shape is summarized in Fig. 4and details can be referred to elsewhere^33,34^. For WSA_REF_ and WSA_m−RAY_ the proximal and distal diameter-calibrated pressure waveforms with their measured flow (for WSA_REF_) and modelled flow (for WSA_m−RAY_) were transformed into their frequency domain components. An impedance analysis ($\:\left|\text{P}\left(\text{j}{\upomega\:}\right)\right|/\left|\text{Q}\right(\text{j}{\upomega\:})|$) was performed to compute the respective magnitude and phase spectrum. Characteristic Impedance (Z_C_) was estimated as the mean of 4 to 10 harmonics of the magnitude spectrum^44^. $\:{\text{P}}_{\text{F},\text{p}\text{r}\text{o}\text{x}}\left(\text{t}\right)$ and $\:{\text{P}}_{\text{F},\text{d}\text{i}\text{s}\text{t}}\left(\text{t}\right)$ were obtained as per Eqs. (6) and (7). Figure 3 provides an overview of the WSA.

**Table 1:** Study participants demography.

Total participants	60
Male participants	36 (60%)
Female participants	24 (40%)
Age range (years)	20–60
Height (cm)	169.8 ± 11.5
Weight (kg)	68.5 ± 8.1
Body Mass Index (kg m^−2^)	24.5 ± 5.1
Heart Rate (bpm)	70 ± 11
Brachial SBP (mmHg)	116 ± 16
Brachial DBP (mmHg)	73 ± 12
Brachial MAP (mmHg)	87 ± 13
Brachial PP (mmHg)	42 ± 6
Carotid Tonometry SBP (mmHg)	99 ± 13
Carotid Tonometry PP (mmHg)	26 ± 4
Peak Systolic Flow Velocity (cm s^−1^)	60 ± 11
End Diastolic Flow Velocity (cm s^−1^)	14 ± 4

### Statistical analysis

The group average values of each computed or measured variable were reported as mean ± standard deviation. The measure of repeatability of any variable was reported as coefficient of variance (CoV), computed as the ratio of standard deviation to mean, expressed as a percentage. Single-factor ANOVA with multiple comparisons between groups was performed to assess whether the mean difference between groups had any statistically significant differences or not. Wherever the test for normal distribution failed for a group, equivalent non-parametric tests such as Kruskal-Wallis were performed as an alternative to ANOVA. The correlation between any two groups was reported using Pearson’s correlation coefficient (r-value). A linear regression analysis was performed to determine the linear trend line between the dependent and independent variables. Bland-Altman analysis between difference and average of to groups or methods was perormed to evaluate the average discrepancy between two methods. Bias and 95% limits of agreement (LoA = bias ± 1.96 × standard deviation) are used to denote the limits of agreement between two methods or groups. A two-tail p-value was used in all the cases to determine the statistical significance of the test. A level of significance (α) of 0.05 was applied to all the tests. A p-valve < α, would reject the null hypothesis of the relevant tests.

## Results

### Study participants characteristics

Table 1summarizes the demography of the study participants. Female participants accounted for 40% of the study population. Female participants were pre-menopausal and non-pregnant. The mean age of the participants was 32 ± 10 years. As per the BMI, 12.3% of the participants were underweight (BMI < 18.5 kg m^[-2^), 40.35% were overweight (BMI > 25 kg m^[-2^), and 12.3% were obese (BMI > 30 kg m^[-2^). Only 5 participants were reported to have stage 1 hypertension and 2 participants of stage 2 hypertension. No participants were under any prescribed medication during or immediately prior to the study period. Pulse waveform classification based on the AIx, revealed 59.70% of the participants were of Type-C, 22.80% of Type-B and 17.50% of Type-A.

**Table 2:** Comparative performance of WSA_m-RAY_ with WSA_REF_.

Reflection Quantification Indices	Group Average Values		Correlation of WSA_m−RAY_ with WSA_REF_
	WSA_REF_	WSA_m−RAY_	
Characteristic Impedance, Z_C_ (mmHg ml^−1^ s^−1^)	1.20 ± 0.55	1.16 ± 0.56	0.85
Carotid Peripheral Resistance (mmHg ml^−1^ s^−1^)	10.63 ± 5.30	12.97 ± 7.41	0.81
Forward Pulse Pressure, ΔP_F_ (mmHg)	27.36 ± 10.24	25.23 ± 7.89	0.89
Backward Pulse Pressure, ΔP_B_ (mmHg)	14.32 ± 6.72	14.47 ± 5.52	0.85
Reflection Magnitude, RM	0.52 ± 0.14	0.58 ± 0.15	0.75
Reflection Index, RI (%)	33.80 ± 6.06	36.13 ± 5.96	0.74
Reflection Wave Transit Time, RWTT (ms)	76.50 ± 27.51	94.79 ± 26.20	0.70

### Reliability of the recorded signals

The dual channel A-mode RF echoes obtained from the in-house developed bi-modal arterial probe had a Signal-to-Noise ratio (SNR) > 30 dB and an attenuation coefficient of 0.7 dB/MHz/cm sufficient for carotid vessel wall identification and tracking. High sampling rates of digitization ensured a tracking resolution of 10 μm for the vessel wall distention and up-sampling, ensured 0.05 ms of temporal resolution for accurate measurement of PTT. The processed diameter waveforms – proximal and distal were continuous and quasi-periodic in nature. The average D_D_ among the study participants was 6.20 ± 0.97 mm, with a mean CoV < 7% and the average ΔD was 0.51 ± 0.16 mm, with a mean CoV < 5%. The tonometry pulse waveforms obtained using the same bi-modal probe, had an SNR > 25 dB and were continuous and quasi-periodic.

The Doppler flow velocity waveform recorded using the duplex-mode of ultrasound imaging system has reliably captured the tri-phasic nature of the carotid blood flow. The peak systolic velocity was 59.94 ± 10.92 cm s^−1^ and end-diastolic velocity was 14.26 ± 4.60 cm s^−1^. The edge-detention based waveform tracing algorithm faithfully extracted continuous doppler flow velocity cycles, preserving the quasi-periodic nature. The doppler flow rate was computed from the vessel cross sectional area and flow velocity, with peak flow rate of 21.66 ± 7.05 ml s^−1^, and the end-diastolic flow rate was 4.50 ± 2.09 ml s^−1^ among the study participants.

**Fig. 7:**
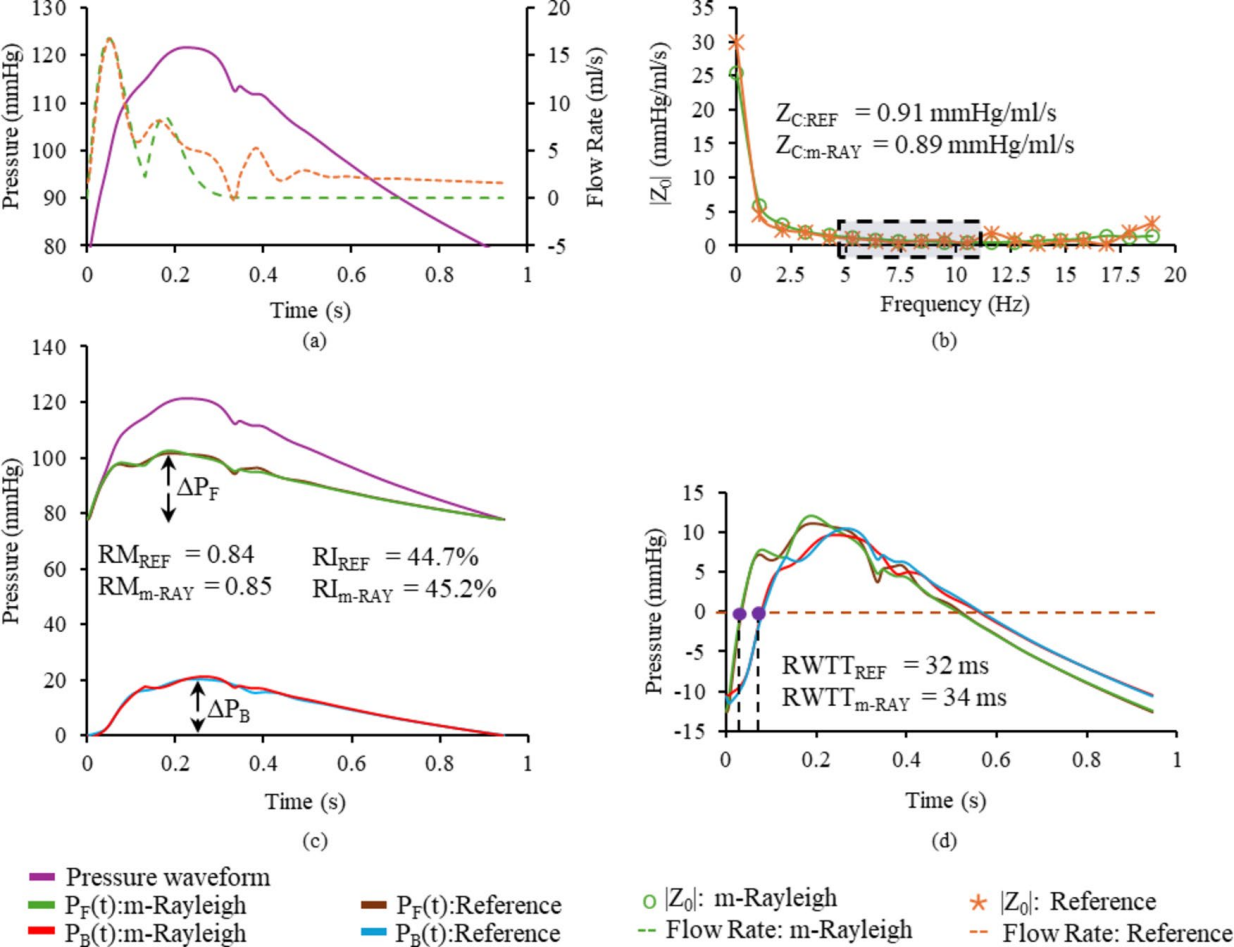
(a) Diameter-scaled and calibrated pressure waveform, measured flow waveform and modelled flow waveform, (b) input impedance magnitude spectrum constructed for WSA_REF_ and WSA_m−RAY_, (c), (d) derived forward and backward pressure waveforms from WSA_REF_ and WSA_m−RAY_, along with their reflection quantification indices (RM denotes reflection magnitude, RI denotes reflection index, RWTT denotes reflection wave transit time).

### Comparative assessment of WSAm-RAY with WSAREF

Table 2 summarizes the average values of the characteristic impedance, carotid peripheral resistance, obtained from the impedance analysis and reflection quantification indices – ΔP_F_, ΔP_B_, RM, RI and RWTT as obtained from WSA_REF_ and WSA_m−RAY_. A sample description of the measured flow waveform, along with modelled flow waveform, with their respective input impedance magnitude spectrum and reflection quantification is depicted in Fig. 7. A strong and statistically significant correlation (*r* > 0.76, *p* < 0.001) was observed between the reflection quantification indices obtained from both WSA.

### Reliability of intra-cycle pressure-dependent variations in local PWV

**Fig. 8:**
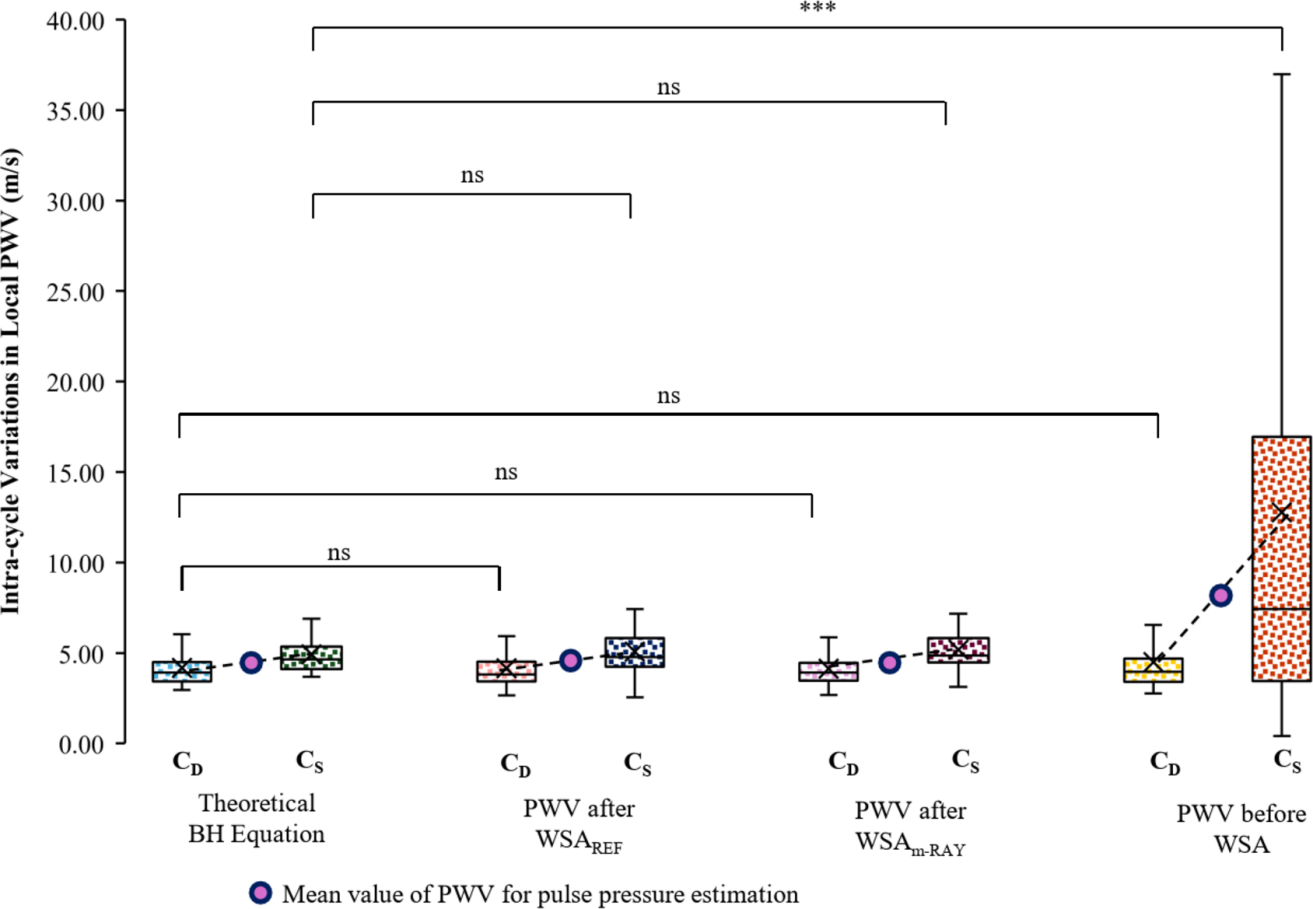
Comparison of PWV at diastole and systole levels, after WSA_REF_ and WSA_m−RAY_, with PWV before WSA and from PWV derived from BH equation (ns: not significant, *p* > 0.05, *** *p* < 0.001).

The average CoV for C_D_: before WSA was ~ 8%, and C_S_: before WSA was ~ 25%. The average CoV was improved to ~ 7% for C_D_: after WSA, and that of C_S_: after WSA was improved to 10%. The group average C_D_: BH was 4.18 ± 1.06 m s^−1^, and C_S_: BH was 4.92 ± 1.12 m s^−1^, with a ΔC of 0.74 m s^−1^. As depicted in Fig. 8, the C_D_: before WSA, C_D_: after WSA_REF_, and C_D_: after WSA_m−RAY_ had statistically insignificant differences in their mean values (*p* > 0.05) with C_D_: BH. The C_S_: after WSA_REF_, and C_S_: after WSA_m−RAY_ also had statistically insignificant differences (*p* > 0.05) with C_S_: BH. The C_S_: before WSA has high variability in measurements (12.77 ± 12.33 m s^−1^) and had a significant difference in their mean values (*p* < 0.001) with that of C_S_: BH.

**Fig. 9:**
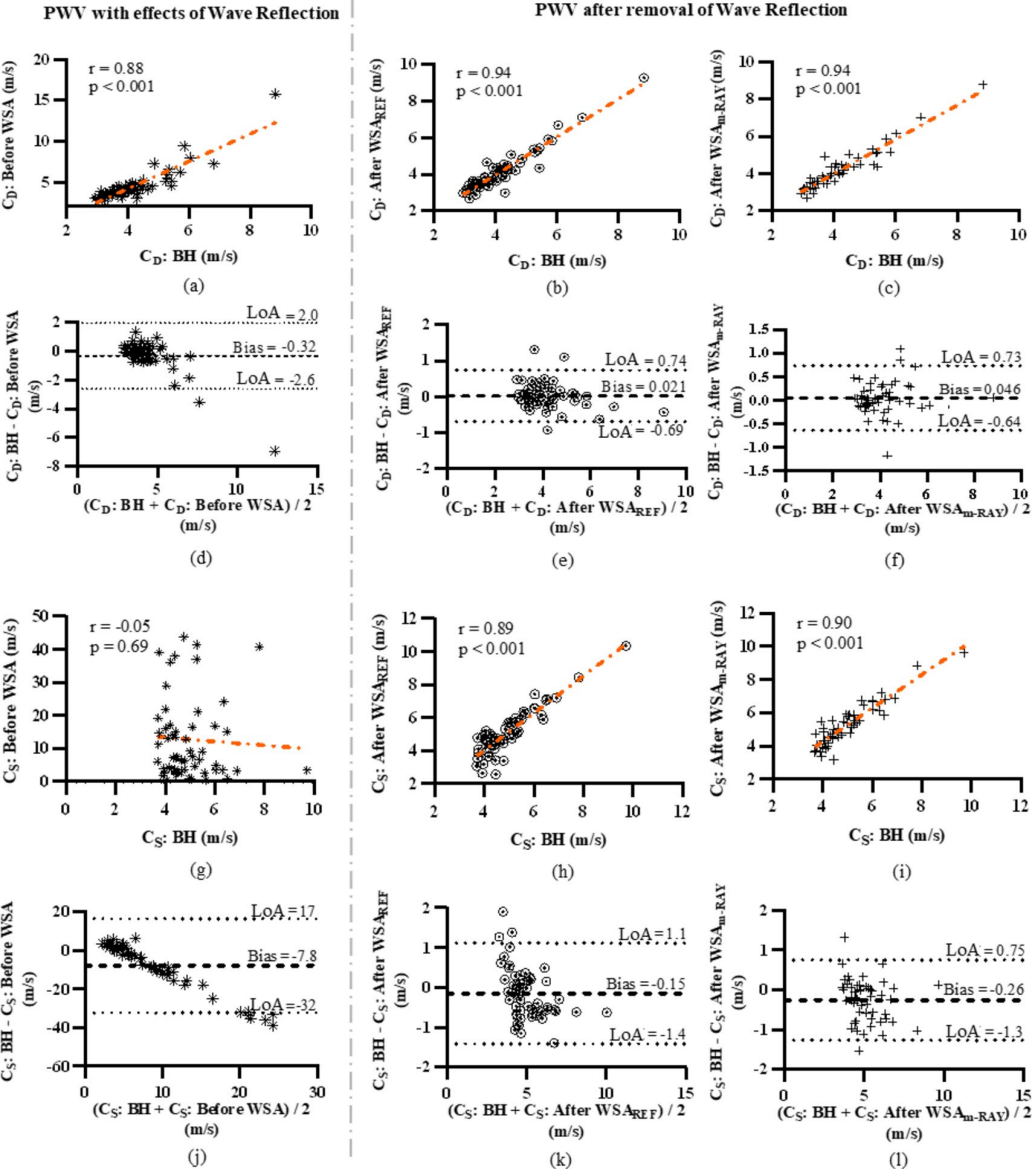
(a), (d), (g), (j) Correlation and Bland-Altman analysis for PWV without performing WSA (with the effect of pulse wave reflections) and (b), (c), (e), (f), (h), (i), (k), (l) after performing WSA (after removal of pulse wave reflections) compared with PWV obtained from BH equation taken as reference. The WSA is computed using measured flow waveform (WSA_REF_) and modelled flow waveform (WSA_m−RAY_). C_S_ and C_D_ corresponds to PWV at systole and diastole within a cardiac cycle.

As depicted in Fig. 9 (a)-(c), there exists strong and statistically significant correlation between C_D_ obtained before (*r* = 0.88, *p* < 0.001) and after WSA (*r* = 0.94, *p* < 0.001) with that of C_D_: BH. For C_S_, as depicted in Fig. 9 (g)-(i) there exists strong and statistically significant correlation (*r* > 0.89, *p* < 0.001) between C_S_ obtained after WSA and C_S_: BH, whereas there was no correlation (*p* = 0.69) observed between C_S_: before WSA and C_S_: BH. The Bland-Altman analysis revealed scattered plot in Fig. 9 (d)-(f), (k), (l) with no clear evidence of any systematic progression of errors for all cases of C_D_ and for C_S_: after WSA with respect to PWV obtained using BH equation. The bias was also observed lower for C_D_ and for C_S_: after WSA, whereas Fig. 9 (j) depicts Bland-Altman plot with significant bias and error trend for C_S_: before WSA.

### Concurrence in the estimated carotid pulse pressure

**Fig. 10:**
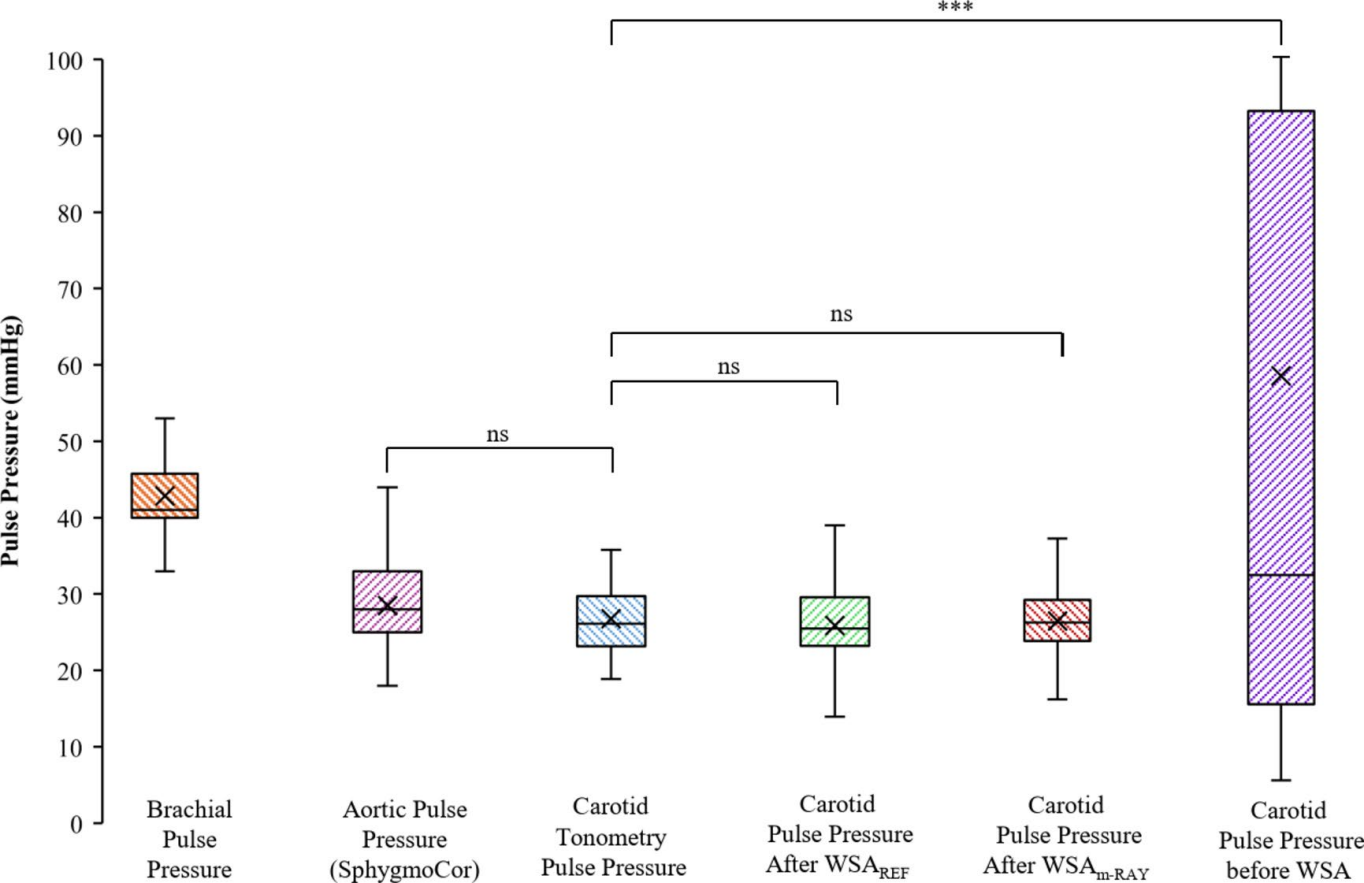
Comparison of pulse pressure obtained from mean PWV, obtained after WSA_REF_ and WSA_m−RAY_, and before WSA, with pulse pressure from carotid tonometry, brachial oscillometery pulse pressure and aortic pulse pressure from SphygmoCor. (ns: not significant, *p* > 0.05, *** *p* < 0.001).

The ΔP obtained using oscillometery device from brachial artery was 42 ± 6 mmHg. The group average carotid ΔP obtained from calibrated tonometry was 26 ± 4 mmHg and the aortic ΔP obtained from SphygmoCor^®^ XCEL was 28 ± 7 mmHg. As depicted in Fig. 10, the mean differences had statistically insignificant differences (*p* > 0.05) for the estimated ΔP obtained using mean values of PWV after WSA and BH equation with that of carotid ΔP obtained from calibrated tonometry, whereas the estimated ΔP obtained using mean values of PWV before WSA, when combined with BH equation yielded higher variability and had a significant difference in their mean values (*p* < 0.001) with respect to carotid tonometry and SphygmoCor^®^ XCEL.

## Discussion

The measurement of PWV largely depends on the accurate description of the waveform morphology and the features derived from it. The reliability of measuring PWV is known to be hampered by the presence of pulse wave reflections. Limited research has been conducted on estimating reflection-free PWV, with most studies relying on loop-based methods which obtains a single average estimate of PWV^27,45,46^. This is first of a study, that computes the ΔC within a cardiac cycle using dual-site PTT measured from forward-travelling pulse waves. In this study, we assessed the accuracy and precision of measuring dual-site PTT-based PWV from measured pulse waves at two points within a cardiac cycle. We repeated the same PTT-based computation after eliminating for wave reflections from the measured pulse waves. As reference, PWV using BH equation was computed from the brachial MAP and DBP-calibrated carotid tonometry and carotid diameter waveforms. The forward-travelling pulse waves were obtained by performing carotid WSA. However, practically, there is a requirement of measuring flow along with the pulse waves to perform WSA. Addressing this challenge, we make use of multi-Rayleigh flow model^33,34^ for performing WSA using a single pulse waveform. Additionally, WSA using measured flow waveform was also performed. The PWV computed from forward-travelling pulse waves obtained via WSA_REF_ and WSA_m−RAY_ were both compared with the reference PWV at diastolic and systolic levels. The method was validated on 60 participants in a pilot study to demonstrate proof of principle. It showed the applicability of using modeled carotid flow with WSA to derive forward-traveling pulse waves. This approach offers both methodological and instrumental advantages over WSA_REF_.

Overall, the results indicate a superior reliability of PWV (C_D_ and C_S_) obtained after performing WSA, on comparing with theoretical PWV. The cycle-to-cycle repeatability was improved marginally (CoV from 8% to 7%) for C_D_. For C_S_, the CoV was improved from 25% to 10%. The mean values of C_D_ and C_S_ after WSA had no statistically significant difference (*p* > 0.05) from the respective mean values of reference PWV. Whereas the C_S_: before WSA had a standard deviation of 12.33 m s^−1^, and no correlation with C_S_: BH (*p* = 0.69). Our observations indicate that C_S_: before WSA is highly corrupted by the presence of wave reflection. Additionally, the carotid ΔP estimated from the mean values of PWV after WSA using BH equation, was in coherence and with no statistically significant difference in mean values (*p* > 0.05) with the carotid ΔP independently calculated from the calibrated tonometry and aortic ΔP computed from SphygmoCor^®^ XCEL, preserving the pulse pressure amplification (PPA) trend from central to brachial artery.

Although several methods exist for measuring local PWV, the transit-time approach is considered the most direct method, that can potentially capture the incremental nature of PWV. However, accurately measuring PWV faces challenges due to the influence of pulse wave reflection, which can distort the pulse morphology and lead to inaccurate results. The accuracy and precision of local PWV heavily relies on selecting the appropriate fiducial point within the systolic phase of the pulse wave. Conventional transit-time approaches often utilize fiducial points near the foot of the pulse wave, such as second derivative maxima, intersecting tangent, or a 10% magnitude threshold, to measure a single value of local PWV corresponding to diastolic pressure^2^. Although, it has been widely accepted that fiducial points near the foot of the pulse wave are relatively free from the influence of wave reflections. However, recent studies have demonstrated that pressure reflections in the vicinity of the systolic foot can corrupt the measurement of local PWV^26,47^. Further, the impact of reflections becomes more evident in the late systolic half, preventing precise measurement of ΔC.

The vessel wall diameter waveforms were recorded using dual-channel focused ultrasound transducers from the bi-modal arterial probe, providing superior tracking resolution (~ 10 μm) and a high frame rate (~ 500 Hz). The data were up sampled to 10 kHz, allowing for the capture of dynamic and instantaneous perturbations at the vessel walls at two sites simultaneously. Ultrasound measurements have higher selectivity to capture the carotid pulse waves, devoid of confounding factors such as effects of surrounding tissue or influence of other vessels as opposed to skin-surface measurements such as photoplethysmogram (PPG) and tonometry. The PPG signals are an average estimate of all the changes in blood volume from all the vessels underneath the sensor whereas tonometry signals are subjected to a hold-down applanation pressure which potentially modulates the vessel wall affecting the pulse morphology itself. The diameter pulse waveforms obtained using ultrasound modality serves as an ideal candidate for computing the PWV as the confounding factors that affect the waveform morphology are minimal. The dual-channel focused ultrasound system used in the study has been previously validated in-vitro with comparable accuracy and repeatability for measuring PWV over a wide range of physiological values from the foot of the pulse wave against high-fidelity invasive pressure waveform recordings from dual-channel pressure-tip catheters and verified in-vivo^48^.

The pressure dependency of the PWV within a cardiac cycle is theoretically laid out by combining the well-established Bramwell-Hill relationship with Hayashi’s empirical pressure-diameter model^2,5,6^. This relationship exhibits an exponential nature, which is attributed to the incremental behavior of the stress-strain curve of the arterial vessel wall as illustrated in Fig. 1 (a)-(c). Specifically, the elastin component becomes the primary load-bearing component at lower pressure levels, while collagen fibers take on this role at higher pressure levels. The C_D_, C_S_ and ΔC obtained from BH equation serves as the theoretical reference values to compare the PWV obtained via other means. As evident from Fig. 8 the C_D_ across methods – before or after WSA are strongly correlated with C_D_: BH, and the mean values are statistically indifferent to C_D_: BH. This is primarily attributed to lower presence of wave reflections, at least as observed in this study population from CCA. There was also a marginal decrease in the CoV of C_D_: after WSA, improving the reliability. These results further confirm, the foot of the measured pulse wave is a reliable fiducial point to measure the PWV but not enough to measure ΔC. The C_S_: before WSA has physiologically unrealistic average value of 12.77 m s^−1^ for CCA with a standard deviation of 12.33 m s^−1^, illustrating the extent to which the pulse wave reflections corrupt the measurement.

The values of C_D_ and C_S_– obtained after WSA and from BH are at par with the values reported in other studies^5–7^. Pulse wave reflection itself is not disadvantageous; in fact, it plays a crucial role in the smooth functioning of the cardiovascular system by ensuring coronary artery perfusion^49^ during the diastolic phase. However, in the presence of stiffer arteries, the wave reflection augments the pulse waveform during the early systolic phase, distorting the measurement of PWV, which relies on waveform features from the systolic phase. The arterial vessel wall acts as the pulse propagation medium for both forward-travelling and the backward-travelling waves. Therefore, the stiffness indices such as PWV derived from forward-travelling waves is an effective technique to address the methodological constraints of measuring PWV and ΔC.

The comparison of ΔP is equivalent to the comparison of estimated PWVs (i.e., average of C_S_ and C_D_) to the reference PWV. The additional comparisons using ΔP at the carotid artery was performed to corroborate previous observations made on PWV. We also observed that the ΔP obtained from the reflection-free PWV lies within the ranges of aortic ΔP that was obtained via SphygmoCor^®^ XCEL and preserves the expected PPA with respect to ΔP at brachial artery. The mean brachial ΔP was 42 ± 6 mmHg for the study population, and the mean carotid ΔP was ~ 26 ± 4 mmHg (refer to Fig. 10) and had a PPA of 16 mmHg from carotid artery to brachial artery. The ranges of PPA were concurrent to what was reported in other studies^50,51^in the range of 10 mmHg to 25 mmHg for central to peripheral PPA. The carotid ΔP is an independent predictor of cardiovascular mortality and morbidity^52^, the results of carotid ΔP obtained from the mean values of PWV after WSA using Eq. (8) with that of the carotid ΔP from tonometry and aortic ΔP from SphygmoCor^®^ XCEL are in coherence. These observations substantiate the results obtained for reflection-free PWV to be reliable and of clinical utility.

The BP varies throughout the arteries due to the complex heterogeneity of vasculature comprising of the elastic central and muscular peripheral arteries. The cBP is observed to have a superior diagnosis for hypertension and early vascular ageing^9–11^. The lack of wide-spread usage in routine clinical examination is limited due to lack of non-invasive technologies for cBP in comparison to auscultation or oscillometery BP devices^12^. The CCA being a direct branch of aorta, acts as a surrogate for central hemodynamics, also has the anatomical advantage for non-invasive sensor placements around the neck. Additionally, arterial stiffness from the central arteries precedes hypertension and metabolic risks especially in the age group 18 to 30 years^53^. With increasing age there exists a clinical consequence of a reversal of the normal arterial stiffness gradient between the elastic central and muscular peripheral arteries in humans^54^. This can lead to pulsatile flow into micro vasculature resulting in end-organ damage. Measurement of arterial stiffness and BP at CCA using local PWV and ΔC are means to develop non-invasive instruments for measuring central hemodynamics and are vital in understanding the pathophysiologic mechanisms of vascular ageing^18^. In recent years, there has been growing interest in exploring the predictive and therapeutic applications of intra-cycle variations in PWV^6,7,16,17^. A population-level study involving 1776 participants provided evidence of the association between intra-cycle variations in PWV and the left ventricular mass index^17^. The incremental PWV, combined with the intima-media thickness of the carotid artery, was associated with improved cardiovascular risk prediction^16^. Additionally ΔC was reported as one of the measurement input for models on central blood pressure in a calibration-free approach^18,19^, enhancing the clinical applicability of the proposed work. With recent advancement in sensing technologies and data acquisition systems, the measurement of ΔC is now made possible to translate to clinical utility and methods proposed in this work, would potentially aid in improving the measurement accuracy. Therefore, ΔC is clinically significant over routinely measured BP from brachial artery for CV risk stratification.

## Limitations and perspectives

While this study provides valuable insights into improving the measurement accuracies of local PWV and ΔC, several limitations should be acknowledged to contextualize the findings. The study participants with low SQ index indicative of a non-parallel artery-probe orientation, are excluded from the study to prevent inaccuracies in the PWV estimation. Often, the fixed distance L of 35 mm between the measurement sites may not always hold true due to the variability in the anatomical placement of the CCA. While we acknowledge the limitation of a fixed distance assumption, the SQ index provides a robust mechanism to ensure that only reliable measurements are considered, mitigating the impact of potential misalignment between the artery and the measurement probe.

As a fundamental limitation of the PTT-based dual site or multi-site methods for measuring local PWV, it is important to explore the the possibility of PPT not being able to track the changes at high BP, especially when leading edge of the pulse wave is getting infinitely sharp. That is, the proposed method would work in case of mild to moderate cases of edge-sharpening effect as observed in the reported study. Researchers who would like to explore these aspects and build upon must consider this potential limitation for measuring PWV between diastole and systole using PTT-based methods.

A volumetric flow rate and a pressure are needed for wave separation analysis instead of a flow velocity. Within a vessel lumen, a flow velocity profile develops and if a flow is constant, a parabolic profile develops, and the volumetric flow rate is directly proportional to the peak velocity of the profile. However, when it comes to pulsatile flow, according to Womersley’s model^55^, a velocity profile constantly changes during a cardiac cycle, and there is not a linear relationship between a peak flow velocity and a volumetric flow rate. Therefore, a flow velocity waveform cannot accurately represent the morphological features of the volumetric flow rate waveform^56^, which should impact the WSA. Therefore, it is suggested in future studies to truly measure the volumetric flow rate using Doppler ultrasound by defining small sample volumes across the lumen and take the weighted average of each velocity or calculating the mean Doppler frequency shift after uniformly insonating the vessel lumen.

Although site-dependent flow measurement is recommended to perform WSA. The ultrasonography machine that was used to acquire flow waveform consists of a linear array probe does not have the spatial resolution to distinguish between the proximal and distal site. Therefore, the measured flow waveform would act as a mean flow waveform for both proximal and distal site. However, the modelled flow using WSA_m−RAY_ is site dependent, as the respective pressure or diameter waveform from the proximal or distal site are used to derive the respective modelled flow for WSA. The modelled flow waveform assumes a close coupling between flow and pressure waveform. The pressure waveform features – healthy or diseased are used to construct the flow model. However, the applicability of the WSA_m−RAY_ is validated on in-silico and in-vivo studies on healthy participants only. Therefore, the study participants recruited are also healthy. The applicability of the model to diseased participants warrants additional investigations. On the application to other peripheral sites using the WSA_m−RAY_, the model performs better estimation of reflection quantification indices for the ones with a non-substantial retrograde flow (such as the carotid artery) rather than the sites with a significant retrograde flow (such as the radial artery). Modelling for such flow profiles involve additional efforts, which are currently not employed. Improvements in the model developed around retrograde flow profiles are in progress and would potentially be applicable to multiple arterial sites.

To be in line with the existing literature on WSA, the proposed method used the classical theory of WSA without any modifications. Since the WSA theory assumes a constant value of Z_C_ to satisfy the water hammer equations and transmission line theory assumptions, modifying the existing theory of WSA using a time-dependent Z_C_ would require a separate investigation. The effect of non-linearity of Z_C_ may enhance the accuracy and precision of the model further.

Ideally the WSA must be performed using the pressure and flow waveform obtained from the same cardiac cycle, which are acquired using instruments that are synchronized to have minimal time-lag between waveforms. However, use of multiple instruments and large form factors of the probe used, often limits the feasibility of simultaneous measurements and studies are compromised with sequential measurements. An underlying assumption of the sequential measurements is that the periodicity in steady state behavior of the hemodynamics. In strict sense, the hemodynamics is assumed to be quasi-periodic, within the small interval of measurement, and the baseline periodicity is continuing until all the measurements are performed. In this study the quasi-periodic steady state of the participants is guaranteed by ensuring a continuous supine posture with no motion artifacts throughout the measurement period and monitoring the bBP at the beginning and end of the measurement protocol. The non-significant deviations in the BP and heart rate ensures the participant had a quasi-periodic state, suitable for sequential measurements. Further epidemiological trials using the proposed method would also explore the clinical significance of local PWV and ΔC as potential prognostic markers for cardiovascular screening.

## Conclusion

In this work, we evaluated the reflection-free PWV and its pressure dependent changes within a cardiac cycle and compared it against theoretical values of PWV near diastole and systole levels. The method involves computing the PTT between two forward-traveling pulse waves obtained from the CCA. The WSA_REF_ was adopted to decompose the pulse wave into forward and backward component. Further a simplified WSA technique that relies on a single pulse waveform (WSA_m−RAY_) was applied to perform the same function, and both the methods of WSA produced statistically insignificant differences in mean values (*p* > 0.05) to reference PWV at diastolic and systolic levels. Whereas the PWV at systolic level without WSA had mean values out of the physiological ranges with a cycle-to-cycle repeatability > 25%. After performing WSA, the repeatability of PWV improved to ~ 7% near diastole and ~ 10% near systole level. Solving the methodological constraints to measure the pressure dependent incremental PWV reliably strengthens the ongoing global efforts on using PWV as clinical marker for cardiovascular risk stratification.

## Data Availability

The in-vivo data that support the findings of this study are available from the Indian Institute of Technology (IIT) Madras, under the guidance of Institute Ethics Committee and are not publicly available. Data are however available from the authors upon reasonable request and with permission of IIT Madras. Dr. Jayaraj Joseph (jayaraj@ee.iitm.ac.in) can be contacted upon with your reasonable request.
